# Monomer release, cytotoxicity, and surface roughness of temporary fixed prosthetic materials produced by digital and conventional methods

**DOI:** 10.1007/s10266-025-01091-8

**Published:** 2025-03-26

**Authors:** Zeynep Sahin, Deniz Ozkan Vardar, Ekin Erdogmus, Semih Calamak, Belma Koçer Gumusel

**Affiliations:** 1 Department of Prosthodontics, Faculty of Dentistry, Lokman Hekim University, Ankara, Turkey; 2 Lokman Hekim University, Vocational School of Health Services, Pharmacy Services Program, Ankara, Turkey; 3 Department of Pharmaceutical Toxicology, Faculty of Pharmacy, Lokman Hekim University, Ankara, Turkey; 4 Department of Basic Pharmaceutical Sciences, Faculty of Pharmacy, Lokman Hekim University, Ankara, Turkey; 5 Department of Pharmaceutical Toxicology, Faculty of Pharmacy, Okan University, İstanbul, Turkey

**Keywords:** CAD/CAM, 3D printing, Cytotoxicity, Temporary restorative materials, HPLC

## Abstract

This study compared surface roughness, monomer release, and, cytotoxicity of temporary fixed prosthetic materials manufactured using the conventional, CAD/CAM milling and 3D printing methods. Disc-shaped samples (2 mm height, 5 mm diameter) were prepared from four materials [polyethyl methacrylate/polymethyl methacrylate (Dentalon Plus-DP), bis-acrylic composite resin (Protemp 4-PT), polymethyl methacrylate CAD/CAM disc (On Dent), and methacrylate-based resin (QuraCROWN Temp)]. Surface roughness was measured with a profilometer; scanning electron microscopy (SEM) was used for surface characterization. Following 24, 72, and 120 h of artificial saliva incubation for the samples, the obtained extracts were evaluated for cytotoxicity by performing 3-(4,5-dimethylthiazol-2-yl)-2,5-diphenyltetrazolium bromide (MTT) test in the mouse fibroblast cell. Monomer release from the test samples was analyzed by High‑Performance Liquid Chromatography. Attenuated Total Reflectance Fourier Transform Infrared Spectroscopy (ATR-FTIR) was performed to evaluate the chemical composition of artificial saliva extracts. Cell viability was assessed by one-way ANOVA, and surface roughness by Kruskal–Wallis and Mann–Whitney U tests. No monomer was detected in artificial saliva for any materials. The FTIR spectroscopy of the extracts did not show any peaks corresponding to these monomer or polymer structures, indicating that no residual monomer or polymer was released into the artificial saliva after exposure to artificial saliva. 3D-printed materials were significantly more cytotoxic than the other three test materials at all time points and dilutions (p < 0.05). The highest cell viability rates were detected in CAD/CAM milling (99.43 ± 3.79) at 24 h and PT materials (100.47 ± 5.31) at 72 h for 1:8 dilution. At 1:4 dilution, except for the DP-3D printing test groups, the other groups show similar cell viability rates with the control group (p > 0.05). Digitally manufactured materials had lower roughness than conventionally produced ones (p < 0.05). CAD/CAM milling and PT materials were the most biocompatible, while 3D-printed material was found to be cytotoxic. CAD/CAM milling and PT materials may offer safe and effective options for temporary prosthetic restorations. Although DP showed acceptable results, it was less effective than CAD/CAM milling and PT materials. Due to their cytotoxicity, 3D-printed materials require further investigation before clinical use.

## Introduction

Temporary restoration is an important and necessary stage of fixed prosthetic and dental implant treatments until the final delivery of the prosthesis [1, 2]. Before a permanent restoration is inserted, these temporary restorations protect both the prepared teeth and adjacent soft tissues. Additionally, they help the patient with fundamental activities while waiting for the final restoration to be applied and the teeth to be prepared [3]. Properly prepared temporary restorations prevent the tooth from returning from its normal position and ensure the maintenance of aesthetic and oral functions such as chewing and speech [4]. Additionally, the materials used in the creation of temporary prostheses must fulfill the mechanical, physical, and biological criteria necessary for use in the oral environment [5].

Computer-aided design and computer-aided manufacturing (CAD/CAM) prefabricated blocks are also available as temporary restoration materials. These materials are composed of either PMMA or resin nanoceramics. They exhibit good mechanical and chemical properties due to being polymerized under controlled pressure and temperature conditions [6]. Additive manufacturing is one more processing technology that has emerged in recent years. Photoinduced liquid resin production is a viable option for the creation of polymer-based crowns and bridges [7].

During prosthetic therapy, temporary restorations are typically utilized for a short duration. On the other hand, there are clinical scenarios in which the use of temporary restorations is prolonged. These situations include intricate oral rehabilitations that necessitate interdisciplinary cooperation and involve orthodontic, endodontic, periodontal, and surgical therapies [3]. As a result, the temporary restorative materials come into extended contact with the gingiva. The interaction between the soft tissues around the prepared tooth and the temporary materials is another important feature [6].

During production, it’s preferable for the monomers found in temporary crown and bridge materials to undergo complete polymerization, forming a network structure ideally. In actuality, though, some of the mixture’s monomers are present unbound because they are not crosslinked into the network [7]. The leaching of residual monomers from dental polymers may lead to irritation, inflammation, and allergic reactions in the oral mucosa [8–10].

To assess the biological performance of dental polymers in clinical settings, conducting biocompatibility studies is essential [8]. Cytotoxicity testing is important in determining biocompatibility [11]. Although 3DP resins have been promoted as biocompatible, there is little research on the subject [12], and there is a lack of information about the chemical components of these resin-based materials and the best manufacturing practices to reduce any potential negative effects [13].

Few studies have explored the correlation between cytotoxicity and the release of residual monomers from temporary fixed restorations [5, 8]. Additionally, there is a lack of research examining monomer analysis in temporary materials, particularly those produced via 3D printing methods, through extract analysis using artificial saliva, and assessing their cytotoxic effects on the L929 mouse fibroblast cell model. Concerning all the available data, the present study aims to investigate the surface roughness, monomer release, and cytotoxicity of L929 cells of temporary fixed prosthetic materials produced by conventional, CAD/CAM milling and 3D printing methods.

The null hypotheses of the study are those (i) the resin extracts obtained after 24, 72, and 120 h of incubation have the same effects on L929 cells viability, or they have the same potential for cytotoxicity (ii) the surface roughness and monomer release of the tested materials will be similar.

## Materials and methods

### Chemicals

Bisphenol-A glycidyl methacrylate (Bis-GMA), triethylene glycol dimethacrylate (TEGDMA), urethane dimethacrylate (UDMA), Methyl methacrylate (MMA) and ethoxylated bisphenol-A dimethacrylate (BisEMA) monomer were obtained from Sigma-Aldrich Co. (St. Louis, MO, USA). 3-(4,5- dimethylthiazol-2-yl)-2,5-diphenyltetrazolium bromide (MTT) were obtained from ThermoFisher (Biotium, Hayward, CA, USA). Dimethyl sulphoxide (DMSO) was purchased from Merck (Darmstadt, Germany). All cell culture materials were from Capricorn Scientific GmbH (Ebsdorfergrund, Germany). All other chemicals were from Sigma-Aldrich (St. Louis, MO, USA).

### Test sample preparation

A power analysis (using the G*Power version 3.1.9.2 package program) was conducted to determine the sample size required to identify a statistically significant difference in the parameters to be measured at three time points (24 h, 72 h, and 120 h) among the four temporary material groups. With an effect size of 0.5, 80% power, and an α error level of 0.05, the minimum sample size needed for the study was calculated to be 9 samples per group, totaling 36 samples. Nine samples were taken for each subgroup.

**Fig. 1:**
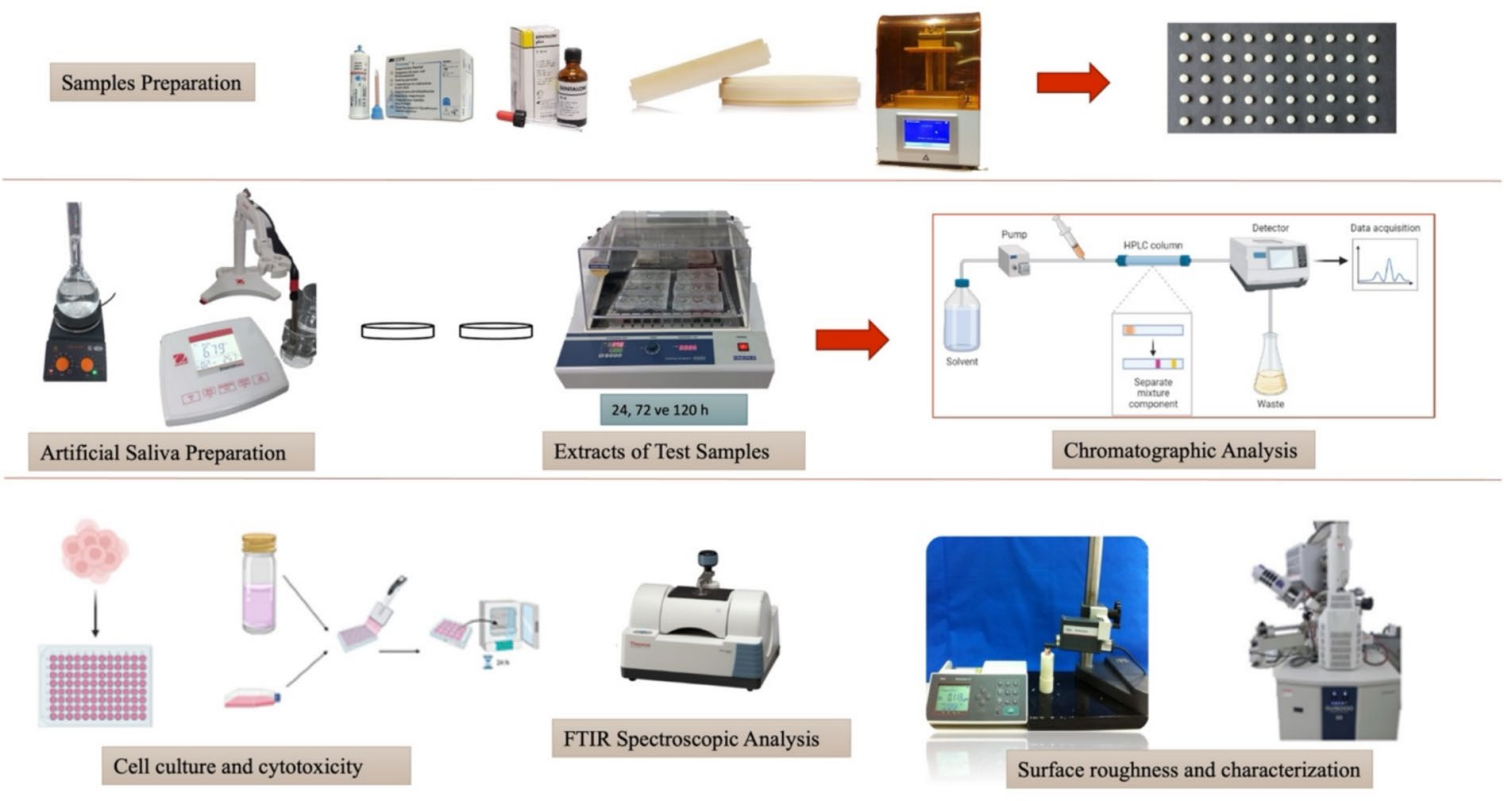
Schematic representation of the experimental design

The materials used in the study, along with their chemical compositions, are detailed in Table 1. Temporary fixed prosthetic test samples produced by conventional methods using a stainless-steel mold with a height of 2 mm and a diameter of 5 mm were prepared according to the recommendations of the manufacturer. Bis-acrylic composite resin-based temporary restoration material (Protemp 4, 3 M ESPE, Seefeld, Germany) is an automatically mixed room temperature chemically polymerized material consisting of two components, a base and a catalyst. Polyethyl methacrylate/polymethyl methacrylate-based temporary material (Dentalon Plus, Heraeus Kulzer, Hanau, Germany) consists of powder and liquid. The powder/Liquid ratio was 2 g/1–1.2 ml, and the liquid was mixed by adding powder, and the mixture was continued until homogeneous. After mixing, the creamy paste was poured into standard molds. To decrease oxygen inhibition and maximize surface smoothness, samples were prepared aseptically between glass plates during polymerization. The polymerization was allowed to complete at room temperature. In the digital subtractive technique, disc-shaped test samples of the same dimensions were designed using a software program (SolidWorks Corporation, Waltham, USA). The obtained data was converted to STL file format. This data in STL format was transmitted to the milling machine (Coritec imes-icore 250i, Onex Dental, Eiterfeld, Germany), and these group’s samples were milled from pre-polymerized CAD/CAM blocks. 3D printing group test samples were produced with a 0° angle and a layer thickness of 50 µm by sending STL data to a printer (Runyes DLP 3D Printer, Ningbo, China). After the post-production 3D test samples were cleaned using isopropanol in an ultrasonic cleaner (Accuretta FreeShape, Ultrasonic Cleaning Unit, Accuretta, Taipei, Taiwan) for 5 min, the post-polymerization process was completed by curing in a UV oven (Acuretta UV Box, Accuretta, Taipei, Taiwan) for 3 min. After finishing with 400, 600, and 800 grit sandpaper (Atlas, İstanbul, Turkey), all test samples were measured with a digital caliper (Mahr, GmbH, Esslingen, Germany), and their dimensions were checked. The experimental design of the study is presented in Fig. 1.

**Table 1:** Temporary fixed materials used in the current study and their chemical contents

Material type	Trade name	Composition	Manufacturer
Bis-acrylic composite resin	Protemp 4	Bis-GMA, UDMA, TEGDMA, Bis-EMA, 50 nm silanized amorphous silica	3 M ESPE, Seefeld, Germany
Polyethyl methacrylate/polymethyl methacrylate	Dentalon plus	Powder: polyethyl methacrylate, polymethacrylate and inorganic fillers Liquid: n-butyl methacrylate, urethane acrylate and ethyl methacrylate	Heraeus Kulzer, Hanau, Germany
CAD/CAM PMMA	On Dent PMMA CAD/CAM Disk	PMMA, pigments	Turkuaz Dental, İzmir, Turkey
3D printing temporary resin material	QuraCROWN	Aromatic methacrylic oligomer Aliphatic methacrylic oligomer Phosphine oxide	Ackuretta Technologies Pvt. Ltd., Taipei, Taiwan

*Bis-GMA* Bisphenol A glycol dimethacrylate, *UDMA* Urethane dimethacrylate, *TEGDMA* Triethylene glycol dimethacrylate, *Bis*-*EMA* Ethoxylated bisphenol-A dimethacrylate, *PMMA* polymethacrylate, *CAD*/*CAM* computer-aided design/computer-aided manufacturing, 3*D* three dimensional

### Extracts of test samples

The artificial saliva was prepared as a 1L solution containing 0.4 g of NaCl, 0.4 g of KCl, 0.0067 g of CaCl_2_, 0.0889 g of NaH_2_PO_4_(H_2_O), 0.005 g of Na_2_S, and 1 g of CH_4_N_2_O [14]. The pH of the artificial saliva was adjusted carefully to 6.7 by adding 0.1 M NaOH when the solution was acidic and 0.1 M HCl when the solution became basic. This adjustment was performed while continuously monitoring the pH with a pH meter, ensuring precise control of the pH level. The surface areas of each of the prepared test samples were calculated to determine the amount of extraction medium required. These test samples were immersed in artificial saliva, the amount of which was determined to be sample surface area/media volume = 3 cm^2^/1 mL as specified in ISO 10993-5 standards [15]. They were then kept on a shaking incubator at 37 °C for 24, 72, 120 h. The extracts obtained from the temporarily fixed materials were filtered through a 0.22-µm membrane and used for cytotoxicity analysis.

### Chromatographic analysis

The test samples were made using the same identical procedure as the cytotoxicity test samples. For the determination of possible residual monomers in extracts from artificial saliva media of test samples, high- performance liquid chromatography (HPLC, Shimadzu Prominence-i Series LC-2030C 3D Plus HPLC/UV detector) technique was used according to a previously reported procedure [16].

HPLC conditions for standard and sample analyses: The monomer standards used were Bis-GMA (512.59 g/mol), TEGDMA (286.32 g/mol), UDMA (≥ 97%, 470.56 g/mol), MMA (98.5%, 100.12 g/mol) and Bis-EMA (452 g/mol). RP-C18 column (4.6 mm internal diameter × 25 cm in length, with a particle size of 5 µm) was used for separation. A gradient mobile phase system consisting of acetonitrile (A) and water (Millipore ultrapure water) was used. All separations were performed with a linear gradient program as follows; 50–80% A from 0 to 15 min and 80–50% A from 15 to 25 min with a flow rate of 0.9 mL/min. After 25 min, the analytical column was reconditioned under the initial conditions for 5 min. The injection volume was 20 µL. The eluants were monitored by the diode array detector from 190 to 800 nm and the chromatograms were extra at 204 nm since all monomers (Bis-GMA, TEGDMA, MMA, Bis-EMA, and UDMA) exhibit significant absorption at 204 nm. All determinations were carried out at 21 °C. Standard monomer solutions of Bis-GMA, TEGDMA, MMA, Bis-EMA, and UDMA (0.05 mg/mL) were prepared in acetonitrile and freshly used.

## FTIR spectroscopic analysis

Attenuated total reflectance Fourier transform infrared spectroscopy (ATR-FTIR) was used to determine the chemical composition of artificial saliva extracts for 3D printing, CAD/CAM milling, DP and PT materials at 24 h, 72 h, and 120 h. The analyzed monomers belonged to polymers (3D printing, CAD/CAM milling, DP and PT) Bis-GMA, TEG-DMA, Bis-EMA, and MMA. All samples were scanned under infrared light with a wave length of 400–4000 cm.^−1^

## Cell culture and cytotoxicity

Mouse fibroblast cells (L-929, ATCC CCL1, Manassas, VA, USA) were cultured in DMEM medium containing 1% Penicillin–Streptomycin, 2 mML-Glutamine, and 10% fetal bovine serum (FBS), first in 25 cm^2^, and then in 75 cm^2^ culture flasks at 37 °C with 5% CO_2_. When the cells reached sufficient density in the culture flasks, they were passaged and plated. The culture flasks were then incubated at 37 °C in an oven with 5% CO_2_. Cells between passages 4 and 6 were used in the experiment to minimize genetic differences in the cell cycle and cell model. Cells were planted with a density of 10,000 cells per 100 μL in a 96-well plate. Following a 24 h incubation period for cell adhesion and proliferation, the extracts were introduced. Subsequently, they were placed in an incubator containing 5% CO_2_ for toxicity assessment.

The cytotoxicity assay using 3-(4,5-dimethyl-2-thiazolyl)-2,5-diphenyl-2H-tetrazolium bromide (MTT) was conducted following the guidelines provided by the International Organization for Standardization (ISO) 10,993–5:2009 [17]. In the first stage of the MTT assay, cells were exposed to extracts from the test samples (different dilutions 1:1, 1:2, 1:4, and 1:8) for 24 h. In the second step, following the extracts were removed at the end of the incubation period, 100 µl of the prepared MTT solution was added to each well and incubated for 2 h. The MTT solution was removed after the incubation period. In the final phase of the experiment, 150 µl DMSO was added to each well and shaken on a shaker for 5–10 min to dissolve the formazan crystals formed by the cells. The cells cultured in a culture medium containing 0.4% phenol represented the positive control, whereas the cells cultured solely in the culture medium served as the negative control. The optical density (OD) of the solution in each well was measured at 570 nm (the formazan absorption peak) on a microplate reader (Synergy H1; BioTek). The viability of the control cells was set at 100%, and the viability of the cells in the test groups was assessed as a percentage (%) relative to these cells. The experiment was repeated three times, and the results were averaged and computed.

Percent cell viability was calculated using the following formula: [17]

$$ {\text{Cell viability}}\left( \% \right) = \left( {{\text{OD of test group}}/{\text{OD of control group}}} \right) \times 100 $$

## Surface roughness and characterization

The test samples’ surface roughness values were determined across three regions (one centrally and two at the peripheries) utilizing a profilometer (Perthometer; Mahr Gmbh, Ingolstadt, Germany). One sample from each group was subjected to scanning electron microscopy (Hitachi SU5000, Tokyo, Japan) analysis at various magnifications (× 1 k, × 2.5 k) following gold plating to qualitatively characterize the surface of this sample.

## Statistical analysis

Analysis of the data was performed using the Statistical Package for the Social Sciences (SPSS) version 22 program (Chicago, IL) and was visualized with Graph iPad PRISM Version 10. Shapiro Wilk test was used to check the assumption of normal distribution. Levene’s test was used to check the homogeneity of variance. Cell viability data were performed by one-way ANOVA with Welch correction. The Games-Howell or Tukey post-hoc test was used to do multiple comparisons. The Kruskal–Wallis test was used to compare more than two independent, non-normally distributed variables for surface roughness data. Additionally, the Mann–Whitney U test was employed to compare two independent variables that did not conform to normal distribution. For statistical significance, a p-value of less than 0.05 was considered.

## Results

### Residual monomer release

**Fig. 2:**
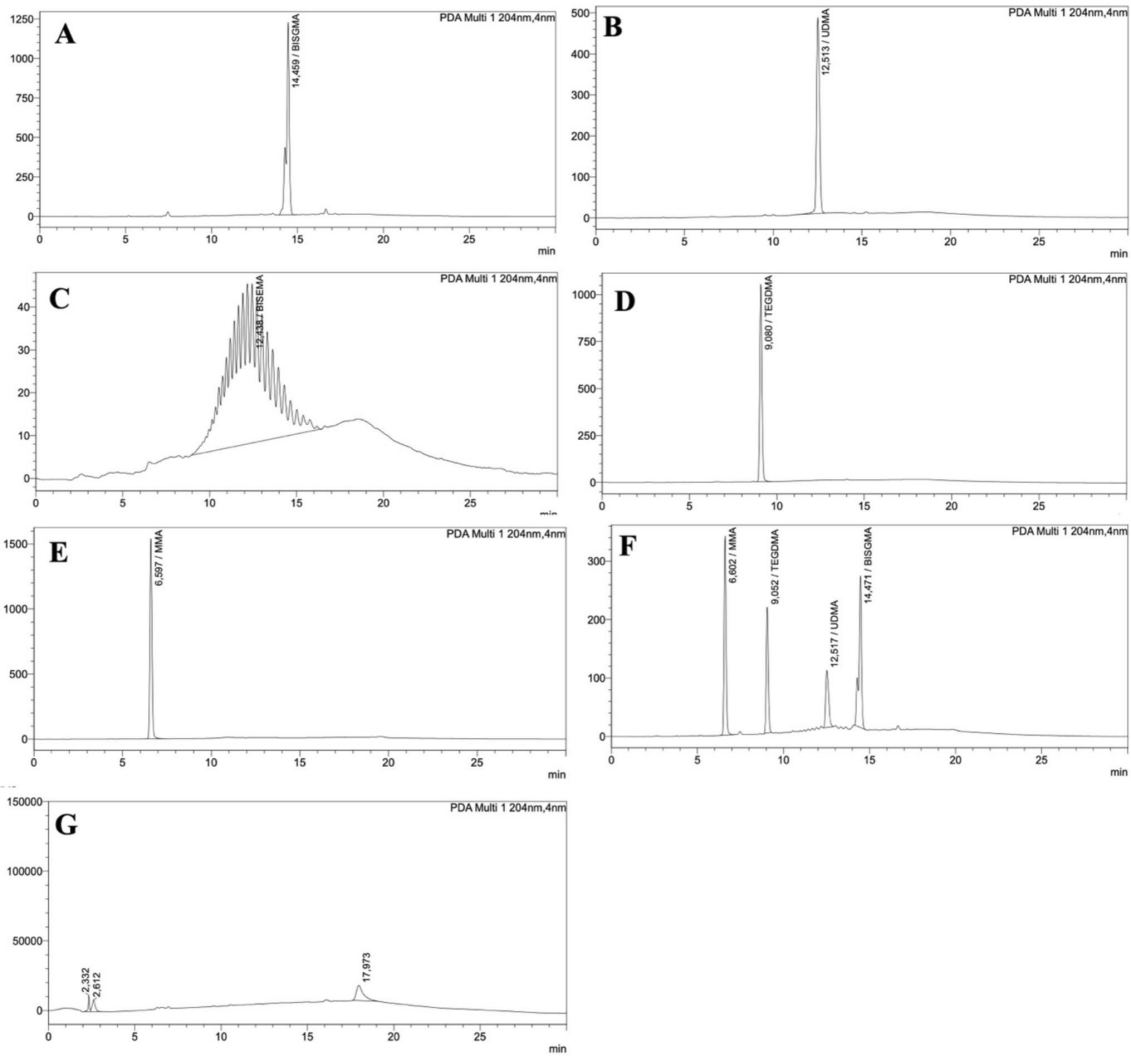
HPLC chromatogram of **A** Bis-GMA, **B** UDMA, **C** Bis-EMA, **D** TEGDMA, **E** MMA reference monomer solution, **F** Mixture of monomer standards and **G** artificial saliva

**Fig. 3:**
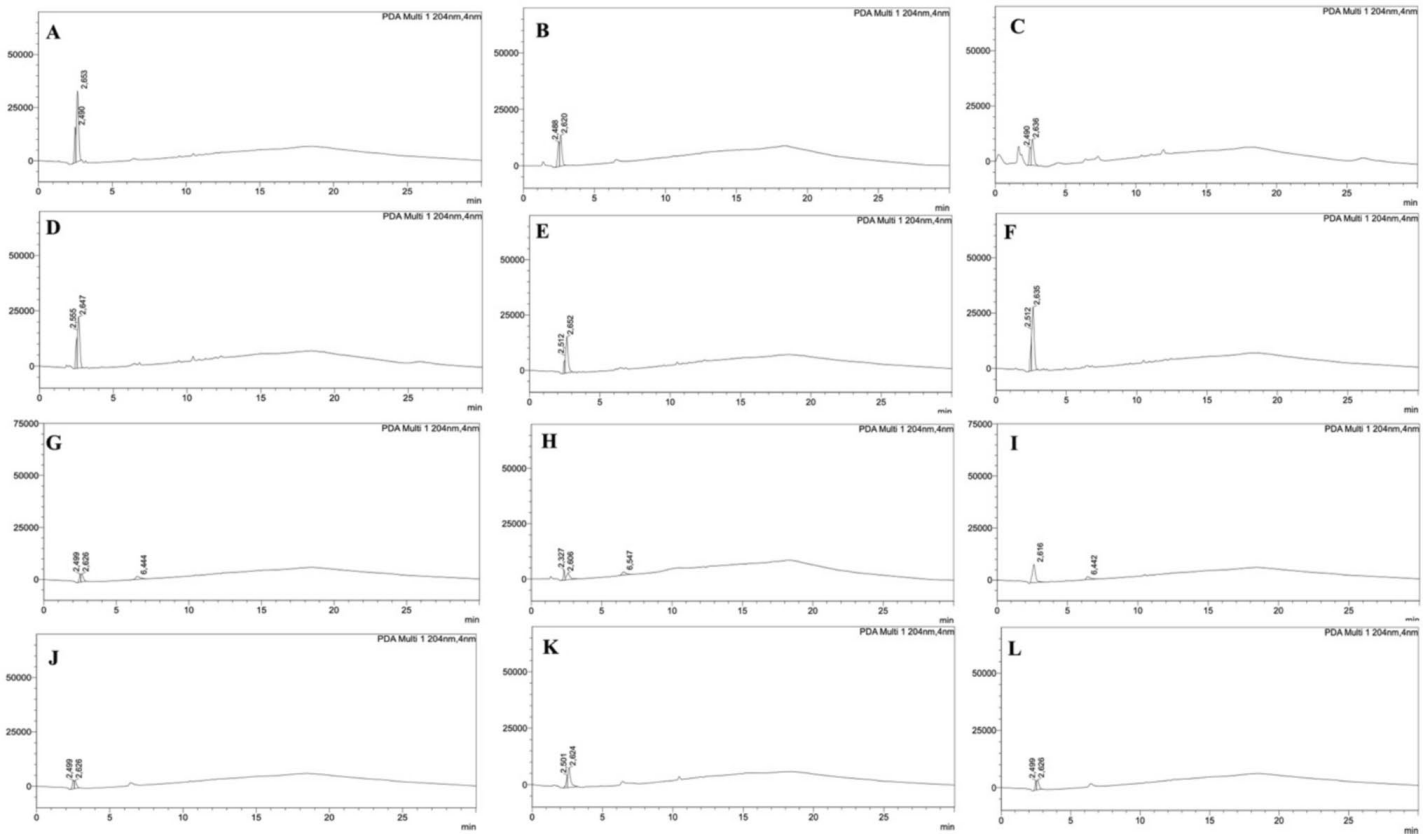
HPLC chromatogram of extract material obtained from CAD/CAM milling **A** 24 h, **B** 72 h, **C** 120 h; Dentalon Plus **D** 24 h, **E** 72 h, **F** 120 h; Protemp **G** 24 h, **H** 72 h, **I** 120 h; 3D Printing **J** 24 h, **K** 72 h, **L** 120 h

The individual peaks and retention times of the standard monomers (Bis-GMA: 14.45 min; UDMA: 12.51 min; Bis-EMA: 12.43; TEGDMA: 9.08 min; MMA: 6.59 min) are presented in Fig. 1. Additionally, in the mixture of basic monomers, the retention times of Bis-GMA, MMA, TEGDMA, and UDMA standard monomers were determined as 14.47, 6.6, 9.05, and 12.51 min, respectively (Fig. 2F) and the samples to be analyzed were evaluated considering these times. Multiple elution peaks were observed in Bis-EMA (Fig. 2C). On the other hand, artificial saliva retention times were determined as 2.33; 2.61; 17.97 min (Fig. 2G). The four different temporary prosthetic materials used in the study did not show any monomer release at 24, 72, and 120 h in artificial saliva (Fig. 3). For all investigated materials at the chosen time points, there was no quantifiable detection of all monomer elution in artificial saliva.

### FTIR analysis results

The infrared spectra of 3D printing, CAD/CAM, DP and PT before and after artificial saliva exposures are shown in Fig. 3. In the infrared absorption spectrum of 3D printing samples, there is an obvious absorption peak between the ranges of 3000–2900 cm^−1^, which is the characteristic absorption peak caused by the stretching vibration of C-H stretching. The carbonyl (C=O) bond associated with the acrylic group is observed at 1720 cm^−1^ and the C=C bond corresponding to the acrylic group is also shown located at 1637 cm^−1^ [18] In addition the FTIR spectrum of Bis-GMA features a peak around 1250 cm^−1^ that is attributed to the epoxide ring enhanced vibration.

It can be clearly seen from Fig. 3 A and D that the characteristic peaks at 1720 cm^−1^ which attiributed C=O stretching vibration for TEG-DMA. The peak at the wavelength of 1635 cm^−1^ is attributed to the stretching vibration of C = C. In the range of 3000–3100 cm^−1^ wavelengths, the aliphatic C-H bonds in the backbone of triethylene glycol are characteristic of the repeating units of triethylene glycol dimethacrylate [19] (Fig. 3A, D). The C=O stretching vibration of Bis-EMA is at about 1720 cm^−1^, indicating the presence of the carbonyl group in the methacrylate group. Spectral fingerprints of aliphatic C-H bonds, typical for ethoxylated groups (broad 3000–3100 cm^−1^ C-H stretching vibration), are also observed in the FTIR spectrum [19, 20] (Fig. 3A, D).

**Fig. 4:**
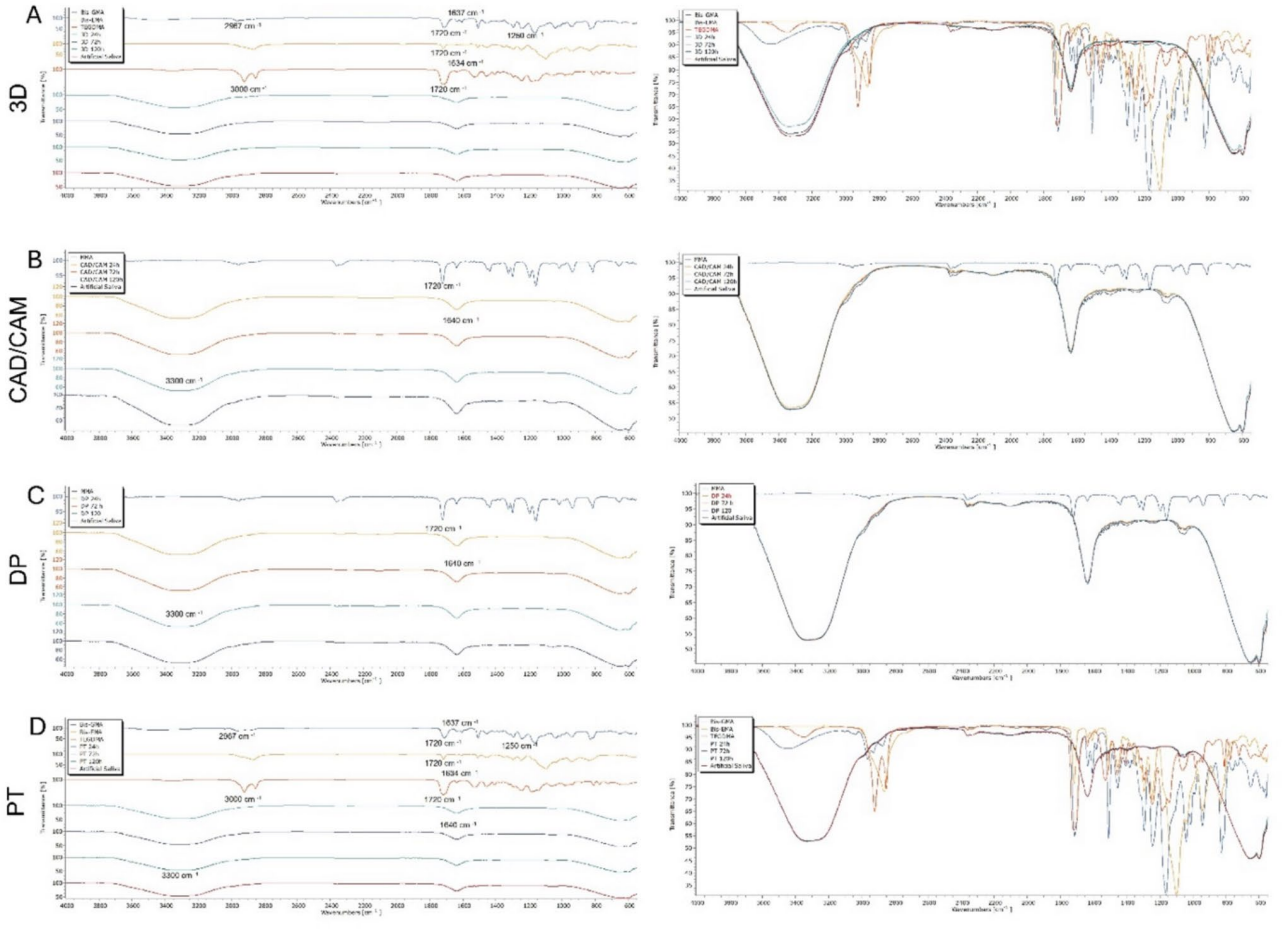
ATR-FTIR spectra of monomers and polymers after artificial saliva exposure. (A) FTIR spectra of 3D samples, showing a comparison between characteristic monomer peaks (Bis-GMA, Bis-EMA, TEGDMA) and spectra obtained after 24 h, 72 h, and 120 h of artificial saliva exposure. (B) FTIR spectra of CAD/CAM samples, comparing the MMA monomer with spectra recorded after 24 h, 72 h, and 120 h of artificial saliva exposure. (C) FTIR spectra of DP samples, comparing the MMA monomer with spectra recorded after 24 h, 72 h, and 120 h of artificial saliva exposure. (D) FTIR spectra of PT samples, showing a comparison between characteristic monomer peaks (Bis-GMA, Bis-EMA, TEGDMA) and spectra obtained after 24 h, 72 h, and 120 h of artificial saliva exposure

For MMA, the presence of C-O stretching vibrations was identified in the range of 1150–1200 cm^−1^, corresponding to ether linkages in the methacrylate structure. The C=O stretching vibration, usually found around 1720 cm^−1^, is one of the strongest peaks in the FTIR spectrum of MMA (Fig. 4B, C). All these results are consistent with the FTIR spectra (Fig. 4) [21].

### Cytotoxicity

Cell viability results of the test materials according to different dilutions at 24 h, 72 h, and 120 h are presented in Fig. 4. When the test materials were compared, they showed similar cell viability rates with the control group (*p* > 0.05), except the 3D printing material for 1:8 dilution at 24 h, 72 h, and 120 h time intervals, and for 1:4 dilution obtained at 24 h. The 3D printing material was found to be more cytotoxic than all other groups (*p* < 0.001).

Following obtaining the 24 h extract, cell viability values at 1:1 dilution (pure) were ranked as Control > CAD/CAM = DP = PT > 3D.

At a 1:2 dilution, CAD/CAM milling showed 89.70 ± 3.94% cell viability, which was similar to the control group, indicating no significant difference (*p* > 0.05). In contrast, Dentalon Plus exhibited 61.03 ± 3.27% cell viability, and the 3D printing material showed 9.13 ± 0.42% cell viability, both of which were significantly more cytotoxic compared to the control (*p* < 0.001). The 3D printing material was the most cytotoxic, with the lowest cell viability among all tested materials (*p* < 0.001). Following extraction for 72 h, CAD/CAM milling at 1:1 dilution showed similar cell viability to the control group (*p* > 0.05). On the other hand, the other groups caused significantly less cell viability than the control group (*p* < 0.001). At a 1:2 dilution, CAD/CAM milling demonstrated the highest cell viability at 80.70 ± 3.00%, while the 3D printing material exhibited the lowest cell viability at 9.73 ± 0.31%. The 3D printing material was significantly more cytotoxic than the other materials tested (*p* < 0.001). At a 1:4 dilution, all groups, except for the Dentalon Plus-3D printing test groups, showed cell viability rates similar to the control group (*p* > 0.05).

When 120 h extract was obtained, all test materials at 1:1 and 1:2 dilution caused significantly lower cell viability than the control group (*p* < 0.05). The 3D printing material was more cytotoxic than the other three test materials (*p* < 0.05).

At a 1:4 dilution, Dentalon Plus demonstrated a cell viability of 84.57 ± 1.65%, while the 3D printing materials exhibited a cell viability of 10.33 ± 0.76%. Both Dentalon Plus and the 3D printing materials were significantly different from the control group (*p* < 0.05). After 24 h of extraction, no significant difference in cell viability was observed between the different dilutions of the 3D printing material (*F* = 0.302, *p* = 0.823).

The undiluted CAD/CAM material exhibited a cell viability of 53.97 ± 7.66%, while the 1:4 dilution showed 81.00 ± 5.51% cell viability, and the 1:8 dilution demonstrated 89.57 ± 6.79% cell viability. The undiluted CAD/CAM material was significantly more cytotoxic than the 1:4 and 1:8 dilutions (*p* = 0.037, *p* = 0.010, respectively). Similarly, the undiluted (pure) group of Protemp material was more cytotoxic than those of the 1:4 and 1:8 dilutions (*p* = 0.045; *p* = 0.025). The undiluted Dentalon Plus material exhibited a cell viability of 66.23 ± 4.31%, and the 1:2 dilution showed a cell viability of 68.17 ± 4.22%. In contrast, the 1:4 dilution demonstrated a cell viability of 84.57 ± 1.65%, and the 1:8 dilution exhibited a cell viability of 87.97 ± 3.12%. Both the undiluted and 1:2 dilutions were significantly more cytotoxic than the 1:4 and 1:8 dilutions (*p* = 0.004, *p* = 0.004, *p* = 0.028, *p* = 0.029, respectively).

When the 3D material was compared according to dilutions after 72 h of extraction, no significant difference was found between different dilutions (*F* = 0.351, *p* = 0.790). In CAD/CAM material, undiluted (pure) was found to be significantly more cytotoxic than other dilutions (1:2, 1:4, and 1:8) (*p* = 0.045, *p* = 0.004, *p* = 0.001). Undiluted, and 1:2 and 1:4 dilutions of DP material were significantly more cytotoxic than 1:8 dilution (*p* = 0.003, *p* = 0.012, *p* = 0.041). Undiluted and 1:2 dilutions of PT material were found to be more cytotoxic than 1:4 and 1:8 dilutions (*p* < 0.001, *p* < 0.001, *p* < 0.001, *p* = 0.005, *p* = 0.003).

**Fig. 5:**
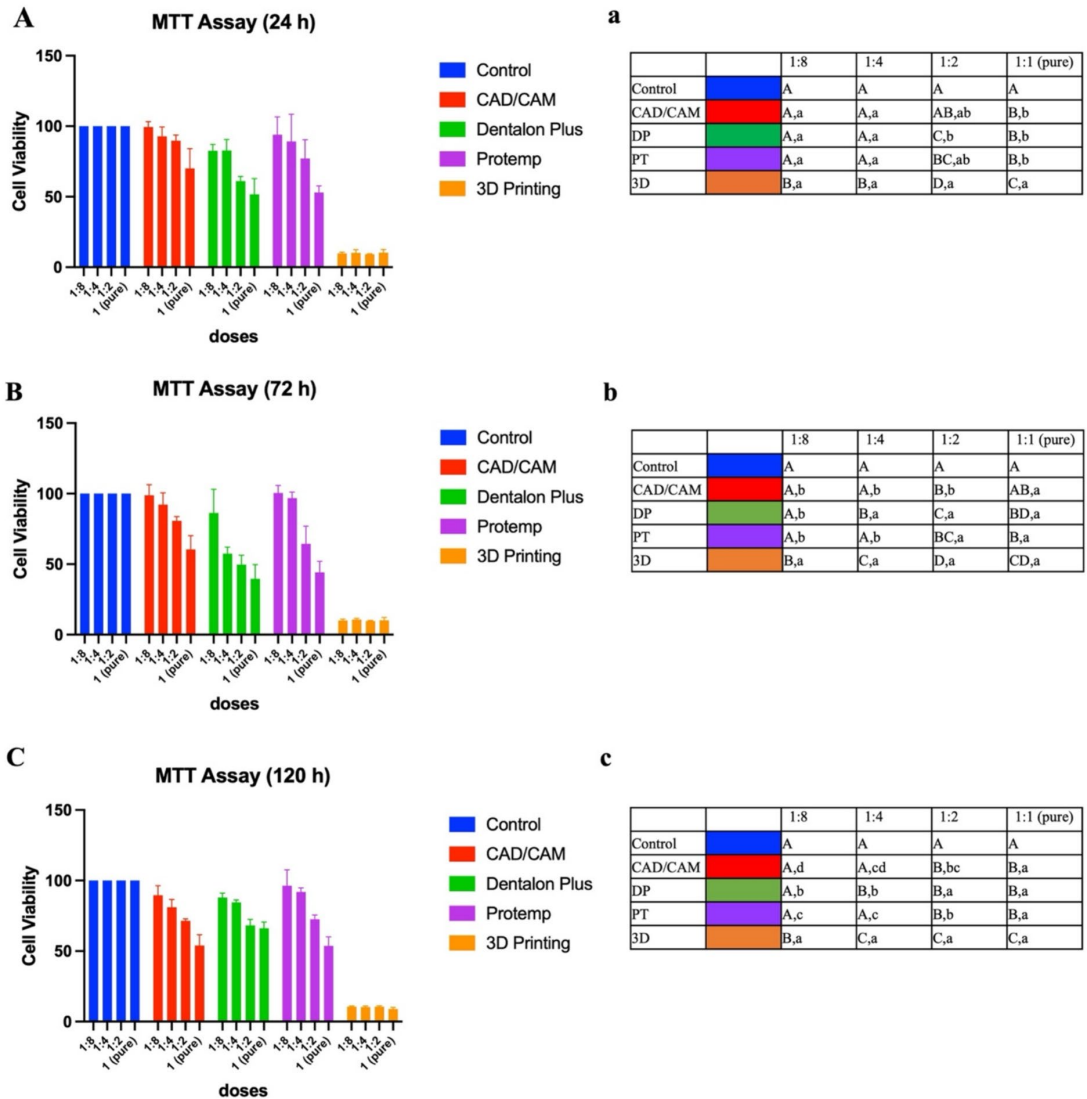
Percent of cell viability according to dilutions of test materials’ extract at (A) 24 h, (B) 72 h and (C) 120 h. **a** Statistical comparison of the test materials after 24 h of extraction. **b** Statistical comparison of the test materials after 72 h of extraction. **c** Statistical comparison of the test materials after 120 h of extraction. p < 0,05, Lowercase letters indicate whether there is a difference among dilutions in terms of time interval same material- between rows, uppercase letters indicate whether there is a difference among test materials in the same dilution—between columns, one-way ANOVA. *DP* dentalon plus, *PT* protemp, 3*D* three-dimensional, *CAD*/*CAM*: Computer-aided design/computer-aided manufacturing

No significant difference was found between different dilutions of 3D material after 120 h of extraction (*F* = 3.089, *p* = 0.090). In CAD/CAM material, undiluted (pure) was found to be significantly more cytotoxic than other dilutions (1:2, 1:4, and 1:8) (*p* = 0.027, *p* = 0.002; *p* < 0.001, respectively). In addition, 1:2 dilution was found to be more cytotoxic than 1:8 dilution (*p* = 0.022). Undiluted and 1:2 dilutions of DP material were more cytotoxic than 1:4 and 1:8 dilutions (*p* = 0.001, *p* < 0.001, *p* = 0.002, *p* = 0.001). In the Protemp material, pure (undiluted) was more cytotoxic than all other dilutions (*p* = 0.037, *p* = 0.001, *p* < 0.001, respectively). 1:2 dilution was also more cytotoxic than 1:4 and 1:8 dilutions (*p* = 0.033, *p* = 0.011 respectively). There was no statistically significant difference between 1:4 and 1.8 dilutions (*p* = 0.858) (Fig. 5).

**Fig. 6:**
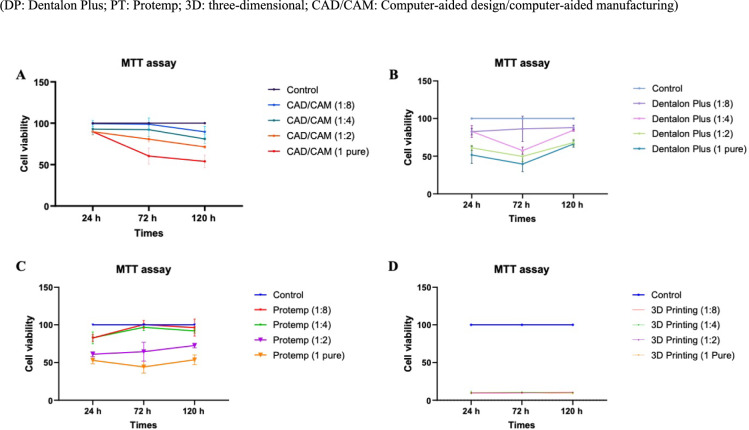
Cell viability percentages of test materials at 24 h, 72 h and 120 h

When the effect of the extracts obtained at different time intervals (24 h, 72 h, and 120 h) on cell viability was analyzed, all test materials at 1:8 dilution showed similar cytotoxic effects within the group according to the time interval (*p* > 0.05). In addition, among the materials, 3D printing materials were significantly more cytotoxic than the other three test materials at all time points for 1:8 dilution (*p* < 0.001) (Fig. 6).

Different extraction times did not cause a significant difference in the cytotoxicity of CAD/CAM, Protemp, and 3D printing materials for 1:4 dilution (*p* > 0.05). However, in Dentalon Plus, 72 h was more cytotoxic than the other two time intervals (*p* = 0.001).

For 1:2 dilution, there is a statistical difference in CAD/CAM test material at all three time intervals (120 h > 72 h > 24 h) (*p* = 0.001). In Dentalon Plus, 72 h was more cytotoxic than 120 h (*p* = 0.009). In intragroup comparison of Protemp and 3D Printing materials, there was no statistical difference in time intervals at 1:2 dilution (*p* > 0.05).

For undiluted pure groups, extracts obtained at different time intervals did not show a statistically significant difference in cell viability depending on the time in CAD/CAM, Protemp, and 3D printing materials (*p* > 0.05). On the other hand, 72 h was more cytotoxic than 120 h in Dentalon Plus material (*p* = 0.026).

### Surface roughness

Digitally manufactured test materials displayed lower surface roughness values compared to conventionally produced ones (*p* < 0.008). The surface roughness values of the test groups produced by the conventional method (bis-acrylic and PEMA/PMMA) did not show a statistically significant difference (*p* > 0.008). However, in the digital methods, a significant difference was observed between the test samples produced by 3D printing and CAD/CAM milling (*p* < 0.008). The highest surface roughness value was observed in the test group produced by the traditional method based on PEMA/PMMA [1.33 (0.64–2.31) μm], while the lowest surface roughness value was observed in the test samples produced by 3D printing [0.20 (0.14–0.56) μm] (Table 2).

**Table 2:** Surface roughness values of the test samples

Test groups	Surface roughness (μm)
Bis-Acrylic Composite resin (Protemp)	0.86 (0.39–1.66)^a^
Polyethyl methacrylate/polymethyl methacrylate (Dentalon Plus)	1.33 (0.64–2.31)^a^
CAD/CAM PMMA	0.33 (0.23–0.38)^b^
3D printing	0.20 (0.14–0.56)^c^
*p*	** < 0.008**

Significant difference is expressed with the different letter–Mann–Whitney U test

### Surface characterization results

**Fig. 7:**
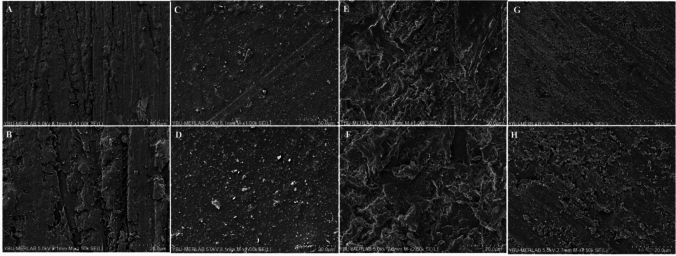
SEM images of the test materials (× 1.00 k, 2.50 k magnifications). **A** and **B** CAD/CAM milling; **C** and **D** 3D Printing, **E**, **F** Dentalon Plus; **G**, **H**: Protemp 4

Dentalon Plus and Protemp materials showed a rougher surface. 3D printing a relatively smooth surface, characteristic pattern, and inorganic fillers were observed. Although CAD/CAM milling is less rough than conventional materials, it was observed that the microscopic surface where the milling bur marks are present during the milling process was rough. In addition, a dense and compact structure was observed. Digitally produced temporary materials had a more homogenous surface than those of conventional ones (Fig. 7).

## Discussion

In this study, we assessed the cytotoxic impact of commonly utilized traditional temporary fixed materials (acrylic and composite-based) in routine dental practice, as well as temporary fixed materials generated through digital technology (CAD/CAM milling and 3D printing), which have gained prominence. Testing was conducted on mouse fibroblast cells, along with assessing residual monomer release from extracts obtained from an artificial saliva medium over durations of 24, 72, and 120 h.

Various dilutions at different time intervals showed different cytotoxic effects of the test materials. After 24 h, 72 h, and 120 h extraction, the 3D test material was found to be more cytotoxic than all other test materials. At 1:8 dilution, all materials except 3D were biocompatible at all time intervals. After 72 h extraction at 1:4 dilution, test materials except DP and 3D printing were biocompatible. Except for the 3D Printing material, cell viability rates differed between dilutions and decreased in a dose-dependent manner. In light of these findings, the first null hypothesis of the study is rejected. The digitally produced test materials had lower surface roughness values than the conventional ones. In addition, the second hypothesis of the study was partially rejected since monomer release from the test materials was not detected in the artificial saliva environment, thus showing similar findings.

Commonly employed resin-based dental materials are recognized to release various monomers following restoration placement, primarily due to incomplete polymerization, but also as a result of erosion and degradation over time [5]. UDMA, MMA, and TEGDMA are the monomers most extensively studied in the literature [7, 8, 22], and are associated with harmful biological outcomes [5].

Bandarra et al*.* [5] identified the release of UDMA in both ProTemp 4 (3.85 μM) and Structur 3 (83 μM), as well as the release of MMA in Tab 2000 (3.47 mM) after 24 h of immersion. In the present study, no monomer release was detected from the test materials after 24, 72, and 120 h of extraction. The difference may be attributed to the medium in which the extract was obtained. Given that the manufacturer’s SDS information does not mention the presence of UDMA in Protemp 4, the Bandarra et al*.* [5] UDMA detection is a remarkable finding. Manufacturers are only obliged to provide information about the main components; therefore, SDSs of dental composites do not always provide comprehensive information about the entire material composition [23].

The absence of any monomer release from the test materials in the present study may be because the resin-based material barely penetrates the polymer network when extracts are obtained in artificial saliva [24, 25]. However, ethanol has been employed by numerous researchers (due to its ability to permeate the material’s polymer network, increasing the spaces between polymer chains and promoting the gradual release of unreacted monomers [26]. An artificial saliva medium was used in this study to obtain extracts because it simulates the oral environment. Additionally, it makes sense to use this kind of solvent because provisional restorations are continuously exposed to saliva once placed. This interaction with saliva extracts and disperses various chemicals from the restorations throughout the oral cavity. Furthermore, using artificial saliva helps to standardize experimental conditions and prevent sample contamination [10].

Berghaus et al*.* [7] used different extraction media [water, ethanol, and ethanol/water (75/25 vol.] from CAD/CAM milling, 3D printing, and self-curing temporary crown and bridge materials and reported that there was no monomer release in the water. This finding is similar to the present study. The low tendency of the monomers to dissolve in water can be attributed to their hydrophobic nature, which is particularly evident in Bis-EMA and Bis-GMA [7].

The complete absence of monomers and polymers in the artificial saliva extracts was proven by FTIR analysis of 3D, CAD/CAM, DP and PT materials before and after 24 h, 72 h and 120 h exposure to artificial saliva. The typical absorption peaks related to the major monomers such as bis-GMA, TEG-DMA, bis-EMA and MMA were identified in the samples before extraction. These included significant peaks such as the carbonyl (C=O) stretching vibration at about 1720 cm^−1^, the C=C stretching vibration at about 1635 cm^−1^, and the aliphatic C-H stretching vibration in the range of 3000 to 3100 cm^−1^. However, the FTIR spectroscopy of the extracts did not show any peaks corresponding to these monomer or polymer structures, indicating that no residual monomer or polymer was released into the artificial saliva after exposure to artificial saliva. This suggests a high degree of material stability and polymerization efficiency, preventing the release of unreacted monomers.

Wei et al*.* [8] reported that the residual monomer released from CAD/CAM polymers (including provisional and prosthetic base polymers) is less compared to conventional ones. The increased cell proliferation observed in CAD/CAM dental polymers has been suggested to result from a reduced release of residual monomers. In the present study, CAD/CAM milling test samples (except pure and 120 h extraction time) were found to be biocompatible. However, no monomer release was detected. The reasons for the difference from the aforementioned study may be related to the chemical contents of the test materials used and the different media from which the extracts were obtained. On the other hand, CAD/CAM milling is more biocompatible than the conventional material (Dentalon Plus), which is similar to the mentioned study.

Atay et al*.* [11] stated in their study that one of the temporary produced by CAD/CAM milling technique (Telio CAD) was not cytotoxic and the other (Vita CAD Temp) showed mild cytotoxic. They reported that this may be due to the difference in chemical composition. In the present study, CAD/CAM milling test samples were found to be biocompatible in all subgroups (except pure and 120 h extraction time). These results partially overlap with the study of Atay et al*.* [11] The difference from the Atay et al*.*’ [11] study is the cell viability rates. The reasons for these are Atay et al. [11] categorized cytotoxicity grading as non-cytotoxic, slightly, moderately, and severely cytotoxic by the millipore filter method. They also used an XTT cell proliferation kit. In the present study, cell viability rates were determined according to ISO standards using MTT assay.

Gonçalves et al*.* [27] investigated the cytotoxicity of two bis-acryl composite resins (Protemp 4, Luxatemp Star) using human gingival fibroblasts. They concluded that these materials do not exhibit cytotoxic effects on human gingival fibroblasts. These findings are similar for the subgroups (except pure) of the Protemp material obtained at all time points in the present study. According to the results of the present study, Protemp material shows high cell viability rates. This can be attributed to the modification of the monomer system, which contains flexible chains instead of the rigid chains of bis-GMA homologs [11].

The primary reason for the cytotoxicity of dental resin materials arises from components that can be released, specifically, monomers and photoinitiators [12, 28]. The decreased cell viability percentage observed in the test samples generated through 3D printing in contrast to conventional and CAD/CAM milled samples may be attributed to the lower monomer-to-polymer conversion ratio in 3D-printed materials compared to other production methods. A range of factors, including the chemical makeup of the resin material, the ratio of photoinitiators, and the application method combined with light exposure, together determine the final degree of monomer conversion [28]. Short post-polymerization durations have been reported to have an adverse effect on the biological compatibility of resins [13].

In a study evaluating the cytotoxicity of temporary and permanent fixed printable materials on human gingival fibroblast cells, it was reported that lower cell viability rates may be caused by Diphenyl (2,4,6-trimethyl benzoyl) phosphine oxide photoinitiator in resins produced in DLP printers [29]. Similarly, in the present study, the 3D-printed resin showed low viability rates. The lower cell viability rates recorded in resins containing photoinitiator may explain the genotoxic and cytotoxic effects [30]. On the other hand, low cell viability may be associated with the release of by-products. As a result, by-products may show some side effects [8, 24].

In our study, cytotoxicity values were measured by obtaining extracts of test samples. Thus, the possibility of synergistic interaction between the substances was also considered. This method more accurately simulates real oral conditions compared to earlier cytotoxic studies that utilized pure substances or combinations of pure substances [5, 31].

The choice of immersion medium is a very complicated issue [32]. One study concluded that TEGDMA monomer can be released in similar amounts in saliva and 75% ethanol. However, for BisGMA, 75% ethanol was found to be more aggressive than saliva [33]. The US Food and Drug Administration (FDA) suggests a 75% ethanol/water solution, which is assumed to be a good food simulator (alcoholic beverages, fruits, and syrup) and hence clinically significant [5]. For this reason, many studies have used ethanol/water mixtures [5, 7, 24]. Nevertheless, the resins soften as a result of the nearly similar solubility characteristics of ethanol and bis-GMA. Particularly for Bis-GMA-based resins and polymers, ethanol/water solutions permeate the polymer matrix and cause irreversible degradation by creating soluble units and enlarging the area surrounding them [34]. Ethanol may not be a laboratory substitute for saliva when monomers other than TEGDMA can be eluted [33]. It was noted that acetone is very aggressive compared to all other storage media and therefore cannot be used as a clinically relevant medium [33]. As extraction solvents, both alcohol-based solutions and cell culture medium were employed; however, it has been demonstrated that albumin binding might result in false negative results, particularly when TEGDMA is detected [35]. More research with various immersion media is required.

Bandarra et al*.* [5] evaluated the cytotoxic effects of urethane dimethacrylate-based (Structur 3^®^), bis-acrylic-based (ProTemp 4^™^), and acrylic resin methyl methacrylate-based (Tab 2000^®^) used routinely in dental practice in a 3T3 mouse fibroblast cell culture model. All of the test materials caused a dose-dependent loss of cell viability, however, only Structur 3^®^ extracts were cytotoxic against 3T3 fibroblasts, with the highest cytotoxic effect (77%) observed at 24 h incubation period, which they attributed to the releasing UDMA monomers. Ulker et al. [36] evaluated the cytotoxicity of two bisacrylic-based (Tempofit Duomix, Protemp 3 Garant) and one UDMA (Revotek LC) based temporary prosthetic materials and found that one of the bisacrylic-based materials (Tempofit duomix) was cytotoxic to L929 fibroblasts. In the present study, 3D-printed test materials were found to be cytotoxic on L929 fibroblast cells.

Cytotoxicity experiments were carried out in accordance with 10,993–5:2009 [17] (except the extraction media), which is recommended for the assessment of the biocompatibility of medical devices used in dentistry, to ascertain if the test materials were cytotoxic against fibroblast cells. The cell viability percentages of all subgroups except CAD/CAM milling 120 h 1:1, Dentalon Plus 24 and 72 h 1:1 and 72 h 1:2 dilution, Protemp material 1:1 (24, 72 and 120 h) and 3D printing are within the threshold value (70%) in ISO standards. In light of these findings, dilution rates and time intervals of the test material are also important in the cytotoxic effect.

In studies examining the cytotoxicity of temporary prosthetic materials, the media and duration of extraction differ from each other. In the studies, 24 h [6, 36–39], 72 h, [8], and 120 h extraction times [5] and cell culture media (DMEM or EMEM, etc.) [1, 5, 8], phosphate buffered saline [39] or artificial saliva [10] were used as extraction media. The reasons for the differences in cell viability rates and monomer release from the studies are that the experimental media and conditions also differ from each other.

Bandarra et al. [5] also evaluated the effect of extracts obtained at different time intervals on the cytotoxicity of the test materials and found that the longer the resin eluted, the lower the cytotoxicity. In the present study, cell viability rates between time intervals at different dilutions differed according to the test materials. No time-dependent change was observed in the cell viability rates of the 3D printing material. On the other hand, In Dentalon Plus, 72 h was more cytotoxic than the other two time intervals (1:4 dilution). For 1:2 dilution, CAD/CAM test material’s cell viability rates were ranked as 24 h > 72 h > 120 h. In Dentalon Plus, 72 h was more cytotoxic than 120 h. It is thought that the reasons for the difference from the study of Bandarra et al*.* [5] may be due to the difference in the cell lines used, the medium from which the extract was obtained, and the chemical composition of the test materials.

Dordevic et al. [10] evaluated the cytotoxic effects of conventionally produced PMMA-based resin extracts on rat macrophage viability under in vitro conditions. They obtained 24, 48, and 72 h extracts in an artificial saliva medium. The highest cytotoxic effect was observed in cells exposed to the highest concentrations (50, 40, and 30%) of the extracts extracted for 72 h. In the present study, for Dentalon Plus at 1:2 and 1:4 dilution, the most cytotoxic time interval was found at 72 h of extraction. In Dentalon Plus material, undiluted (pure) and 1:2 dilutions were more cytotoxic than 1:4 and 1:8 dilutions. These results are similar to the previously mentioned.

In the current research, the groups that were digitally generated exhibited substantially lower surface roughness than the conventional groups. These results were found to be consistent with Giti et al. [1]. Based on the surface roughness result of this study, it can be concluded that production methods affect the surface properties of the test materials and that digitally produced temporary materials may cause less discoloration and plaque accumulation than the conventional method. The surface roughness results are supported by the SEM images. The conventionally produced test materials exhibit a rougher structure than the digitally produced ones is similar to the SEM findings of Giti et al*.*[1]. Greater porosity and air bubbles are also observed in SEM images because it is challenging to achieve a relatively homogeneous mixture due to manual mixing in materials produced by the conventional method.

Shim et al. [39] evaluated the response of human gingival fibroblasts to temporary materials with different production methods and various chemical compositions and also examined the surface roughness of the materials. PMMA and bisacryl-based materials were reported to be less cytotoxic in HGF cell lines than PEMA ones. CAD/CAM milling materials have been recommended for use due to greater cell adhesion and prevention of residual monomer. PEMA and PMMA-based materials were found to be rougher than the bisacryl and CAD/CAM milling groups. In the present study, CAD/CAM milling showed a higher cell viability rate than Dentalon Plus at 24 h extraction and 1:2 dilution. This finding is similar to Shim et al. [39]. When surface roughness results are evaluated, similarly to Shim et al. [39], the CAD/CAM milling shows a smoother surface than Dentalon Plus. However, there was no difference in surface roughness between the conventional materials in the present study. The degree of polishability and smoothness is dependent on several factors, including the material’s inherent chemistry, the initiator, the makeup of the resin matrix, the presence of filler particles, and their size and distribution [40]. In the present study, Ra values ranged between 0.20 and 1.33 μm. These values were equal to or above the Ra threshold of 0.2 μm reported by Bollen et al. [41] but below the clinical undetectability limit of 10 μm defined by Kaplan et al*.* [42].

Chen et al. [38] evaluated the cytotoxicity of two resins for interim restorations (AA TEMP; Enlighten Materials and C&B; NextDent), which were printed using a DLP system and subjected to various post-polymerization processes. The researchers performed the cytotoxicity assay in the L929 mouse fibroblast cell model after collecting the extract for 24 h at 37 °C in cell culture medium. They found that resins not subjected to the post-polymerization procedure exhibited a reduction in cellular metabolism of over 70%. However, post-polymerization times of 1, 5, 10, 15, and 30 min resulted in only a minimal reduction in cellular metabolism. The authors indicated that the absence of post-polymerization left the resin surface unsealed, which allowed toxic substances from the inside of the material to migrate into the culture medium. With longer post-polymerization times, the surface seal improved, leading to enhanced cell viability [38]. In this study, the lower cell viability rates observed compared to the aforementioned study may be attributed to the power and parameters of the device used for post-polymerization, as well as the resin contents used. In the current study, the test samples produced by 3D printing were subjected to post-polymerization for 3 min, and considering the cell viability rates, this time should be increased. Additionally, it is essential to investigate the ideal production protocol to minimize the potential negative effects of 3D-printed temporary materials. It can be inferred that post-processing steps such as additional light curing and washing can improve the biocompatibility of printable materials.

Limitations of this study are the use of media that mimic the oral environment but also the need to incorporate thermal, chemical, and bacterial conditions to assimilate in vivo conditions, even when using human saliva. To simulate saliva flow in the mouth, the continuous change of the immersion medium must also be taken into account. In addition, it should be considered that mechanical effects on the surfaces of the temporary materials during intraoral service may affect monomer release. More studies are needed that can mimic clinical conditions by incorporating these factors into experimental conditions. On the other hand, further development of analytical methods using a combination of HPLC and mass spectrometry could provide valuable information on the identification of by-products from temporary materials.

Within the limitations of this study, the following conclusions can be drawn:

Although no residual monomer was detected in 24 h, 72 h, and 120 h extracts of temporarily fixed materials with different chemical contents in an artificial saliva medium, temporary materials produced by 3D printing were found to be cytotoxic in the fibroblast cell culture model.

Manufacturing techniques impact the surface characteristics of the test materials, with digitally produced test materials exhibiting lower surface roughness values compared to those produced by conventional methods.

### Clinical relevance

CAD/CAM milling and PT materials may offer safe and effective options for temporary prosthetic restorations. Although DP showed acceptable results, it was less effective than CAD/CAM milling and PT materials. Due to their cytotoxicity, 3D-printed materials require further investigation before clinical use. Additionally, digitally produced temporary materials may result in less discoloration and plaque accumulation compared to those of conventionally produced.

## Data Availability

All data regarding surface roughness, monomer release, and cytotoxicity of temporary fixed prosthetic materials produced by digital and conventional methods, which support the findings of this study, are included in this paper. These data are not publicly accessible but can be obtained from the corresponding author upon request.
